# Pharmaceutical Advertising and Public Perceptions in Saudi Arabia

**DOI:** 10.3390/pharmacy12060159

**Published:** 2024-10-23

**Authors:** Mohammed A. Alnuhait, Hana A. Althobaiti, Meshari H. Alharbi, Raef A. Alahmadi, Yasser E. Althubaiti, Abdulrahman A. Alsaedi, Abdullah S. Alshammari, Mahmoud E. Elrggal, Mohammed A. Alrashed, Mohamed A. Albekery, Abdullah A. Alhifany, Abdulmalik S. Alotaibi

**Affiliations:** 1 Pharmaceutical Practices Department, College of Pharmacy, Umm Al-Qura University, Makkah 21955, Saudi Arabia; hathobaiti@uqu.edu.sa (H.A.A.); s441001812@st.uqu.edu.sa (M.H.A.); s441001910@st.uqu.edu.sa (R.A.A.); s441000224@st.uqu.edu.sa (A.A.A.); asshammari@uqu.edu.sa (A.S.A.); aahifany@uqu.edu.sa (A.A.A.); assalotaibi@uqu.edu.sa (A.S.A.); 2 Pharmacology & Toxicology Department, Faculty of Medicine, Al Qunfudah, Um m Al-Qura University, Makkah 21955, Saudi Arabia; merggal@uqu.edu.sa; 3 Department of Pharmacy Practice, College of Pharmacy, King Saud bin Abdulaziz University for Health Sciences, Prince Mutib Ibn Abdullah Ibn Abdulaziz Rd, Riyadh 11481, Saudi Arabia; alrashidm@ksau-hs.edu.sa; 4 King Abdullah International Medical Research Center, Riyadh 11481, Saudi Arabia; 5 Pharmaceutical Care Department, King Abdulaziz Medical City, National Guard Health Affairs, Riyadh 11426, Saudi Arabia; 6 Department of Pharmacy Practice, College of Clinical Pharmacy, King Faisal University, P.O. Box 400, Al-Ahsa 31982, Saudi Arabia; malbekery@kfu.edu.sa

**Keywords:** perceptions, awareness, pharmaceutical advertising, medication promotion, Saudi Arabia, Middle East

## Abstract

Introduction: As the pharmaceutical advertising landscape evolves with digital advancements, this study examines public awareness and perceptions of medication advertisements in Saudi Arabia. It focuses on the effects of regulatory frameworks and evaluates how they influence public understanding and attitudes toward these advertisements. Method: A cross-sectional study was conducted using an electronic survey in Saudi Arabia in December 2023. The survey was distributed on social media platforms and reached a diverse sample of 440 participants. It covered public perception and attitudes toward drug advertisements, knowledge of regulatory laws, and preferences regarding advertising mediums. Results: Out of the 440 participants in the study, who were primarily employees with bachelor’s degrees, there was a clear awareness of drug advertisements. The average age of the group was 33 years, and a significant portion (71.1%) held a bachelor’s degree, with 51.1% being employed. The findings revealed that 25.5% of participants frequently noticed drug ads, while 22.7% saw them very often. Although many found the ads informative, there were significant concerns about unrealistic expectations and the risk of overmedication; 89.8% believed the ads set unrealistic expectations about the effectiveness of medications. Additionally, 60.7% thought that celebrity endorsements might mislead the audience, and 91.1% felt that ads should provide more detailed information about potential risks and side effects. Regarding preferred advertising platforms, mobile apps and websites were favored (47%), followed closely by social media (46.4%). A striking 93.2% of participants believed that drug ads on social media should be subject to stricter regulations, and 96.4% wanted more proactive monitoring of online advertising. Many also reported using other sources, such as medical review sites, to verify medication information. Conclusions: Pharmaceutical advertising in Saudi Arabia must balance ethical transparency with educational value. The influence of digital platforms underscores the necessity for stricter regulation and accurate information dissemination. A collaborative approach is essential to align advertising practices with public health interests and regulatory standards.

## 1. Introduction

Pharmaceutical advertising plays a crucial role in the healthcare industry, particularly as it shifts from targeting healthcare professionals to reaching consumers directly. Historically, drug advertisements were aimed primarily at healthcare professionals, but this focus has shifted significantly towards direct-to-consumer advertising (DTCA) over the past decade [1,2]. This transition has allowed pharmaceutical companies to directly engage the public, boosting medication sales and usage [3,4]. However, this shift has raised concerns about its potential negative impact on public health outcomes and increased drug expenditures, especially in regions where DTCA is restricted or not fully addressed by public policy [5]. Countries like the United States and New Zealand permit DTCA, including product claims, whereas many other countries have stringent prohibitions [6,7,8]. In Saudi Arabia, the Saudi Food and Drug Authority (SFDA) imposes stringent rules on drug advertising. Direct-to-consumer ads for prescription medications are prohibited; these ads can only be directed to healthcare professionals through scientific channels and publications. Advertising is permitted only for non-prescription drugs, and this follow strict guidelines set by the SFDA [9,10]. Saudi Arabia has rigorous regulations on online promotions for non-prescriptive pharmaceuticals, enforced by the Saudi Food and Drug Authority (SFDA), ensuring strict control over advertising content and practices [9,10]. SFDA enforces strict regulations on online promotions for non-prescriptive pharmaceuticals, requiring all advertisements to undergo approval to prevent misleading claims, ensure full disclosure of risks, and align with cultural values. Advertisers must also adhere to specific requirements when using online platforms and social media, including obtaining explicit approval and avoiding any unapproved content or claims. These regulations aim to simplify complex medical information and ensure credibility through reliable references. Despite these efforts, there remains a gap in understanding how these regulations impact public perceptions and attitudes towards drug advertisements. While regulations are designed to protect consumers, there is limited exploration of how these advertisements influence public views and behaviors, particularly on digital platforms. Social media, for instance, has become a prominent tool for pharmaceutical marketing, with companies engaging influencers to promote products directly to consumers [11,12]. This shift towards digital marketing challenges traditional regulatory frameworks and highlights the need for a deeper understanding of how these advertisements are perceived by the public [5,13]. This study seeks to address this gap by examining public awareness and perceptions of medication advertisements in Saudi Arabia. It will explore how the regulatory environment influences these perceptions and attitudes, providing insights into the effectiveness and ethical considerations of pharmaceutical advertising in the digital age.

## 2. Methods

A cross-sectional survey was conducted to explore the perspectives of Saudi citizens and residents on pharmaceutical advertising, focusing primarily on both prescription and non-prescription drugs. This online survey took place in December 2023 and reached various regions across Saudi Arabia. The questionnaire was distributed through social media platforms (Facebook, X, Linkedin, etc.) and email invitations. The main objective was to evaluate public awareness and attitudes toward drug advertisements. To ensure our findings were statistically valid, we calculated the sample size needed based on a 95% confidence interval and a 5% margin of error, considering the national population of around 32 million [14]. This calculation showed that a sample of 385 participants would provide an adequate representation of the Saudi population for our study. We selected participants using convenience sampling, targeting Saudi citizens and residents aged 18 and older. The survey was designed to be accessible online, allowing participants to engage with the questionnaire through a provided link. To develop and validate the questionnaire, we conducted a thorough literature review and consulted experts in drug advertising and pharmacy [15,16]. This validation process included pilot testing, where we assessed face validity and internal validity to enhance clarity and relevance. Feedback from 10 participants during the pilot phase led to significant improvements in the survey’s design and content, ensuring it accurately reflected community opinions on drug advertising.

### 2.1. Questionnaires and Data Collection

The survey questionnaire was crafted to cover a broad spectrum of topics, comprising 35 items organized into seven sections. Section A collected demographic details from participants. Section B assessed their awareness of drug advertising and their understanding and perceptions of it. Section C delved into the different types of drug advertisements. In Section D, we explored participants’ opinions on the regulations governing drug advertising. Section E identified factors that influence engagement with drug advertising programs. Section F tackled the ethical aspects and the impact of drug advertising on the community. This comprehensive structure allowed us to gather extensive and relevant data on drug advertising practices in Saudi Arabia. This study received approval from the Institutional Review Board (IRB) at Umm Al-Qura University, under approval number HAPO-02-K-012-2023-10-1842, on 29 October 2023.

### 2.2. Statistical Analysis

Descriptive statistical methods were utilized to analyze the survey data. For categorical variables, frequencies, and percentages were determined, whereas means and standard deviations were calculated for continuous variables. To enhance the clarity of data interpretation, the findings were displayed through tables and graphs. Data analysis was conducted using statistical software SPSS (version 25.0, Armonk, NY, USA). Categorical data were analyzed using chi-square tests, while T-tests were applied for continuous variables with distributions approximating normality. In cases where normality assumptions were not met, the nonparametric Mann–Whitney U-test was employed. A *p*-value < 0.05 was considered statistically significant.

## 3. Results

Table 1 presents the demographic details of our participants. The participants, comprising mostly employees with bachelor’s degrees, had an average age of 33 years. More than half were female (51.1%), while 48.9% were male. A significant majority (96.6%) were Saudi nationals, with most hailing from Makkah province (85.2%). In terms of marital status, 46.6% were single, 46.4% were married, 3.8% were divorced, 0.9% were widowed, and 2.3% preferred not to disclose their status. Educationally, 71.1% had a bachelor’s degree, and 18.9% had completed high school. Employment status revealed that 51.1% were employed, 29.1% were students, 12% were unemployed and seeking work, 5% were retired, and 2% were self-employed. Regarding family income, 42% reported earning between SAR 6000 and 15,000; 20.7% earned between SAR 15,000 and 30,000; 17.3% had less than SAR 6000; 3% earned between SAR 30,000 and 50,000; and 1.1% earned more than SAR 50,000.

Table 2 highlights how participants encountered drug advertisements. About 25.5% frequently saw these ads, and 22.7% saw them very often. Many participants found the ads informative, but there were concerns about unrealistic expectations and overmedication. Specifically, 89.8% felt that ads set unrealistic expectations about how effective medications are. Additionally, 60.7% believed that celebrity endorsements can mislead viewers, and 91.1% thought that ads should provide more detailed information on risks and side effects. Participants preferred seeing ads on mobile apps and websites (47%) and social media (46.4%), while billboards (37.5%) and TV/radio (23.6%) were less favored. Magazines and newspapers were the least preferred (10.7%).

Table 3 explores participants’ opinions on drug ads and their reasons for these views. Table 4 shows that many participants were aware of FDA regulations on drug advertising, but 82.3% thought it was permissible to advertise medicines according to the Saudi Food and Drug Authority’s regulations. The credibility of the source was seen as the most important factor in evaluating pharmaceutical ads (78.2%), followed by information on side effects and risks (55.7%) and content quality (43.4%). Celebrity influence (14.8%) and ad design (14.5%) were rated as less important. About two-thirds (67.7%) preferred ads that featured success stories or real-life experiences. To improve pharmaceutical advertising, 68.2% suggested a greater focus on medical and scientific information, 59.8% wanted more awareness about safe medication use, 48.6% called for better transparency about risks and side effects, 40.2% supported discussing non-pharmacological treatment alternatives, and 36.1% preferred real-life examples. Only 15.5% felt that stricter rules were necessary. Regarding social media’s role, 46.6% thought it plays a significant role in directing attention to drug ads, while 30.2% saw it as having a mediating role. About 43.2% of participants lacked confidence in the information provided in social media ads, and 15.2% had negative experiences with medications promoted online. To improve public understanding of drug advertising and safety, participants suggested using simple language (75.5%), engaging in social media awareness campaigns (68.9%), organizing interactive sessions (53%), and showcasing safe, inspiring medication success stories (34.5%). Most participants (93.2%) believed that laws for social media drug ads should be stricter, and 96.4% thought regulatory bodies should more actively monitor online ads. Additionally, 78.6% reported using other sources, like medical review sites, to verify medication information before use. No statistically significant relationships were reported between the various factors examined and participants’ perceptions of pharmaceutical advertising.

## 4. Discussion

This study provides a comprehensive view of public perceptions and the impact of medication advertisements in Saudi Arabia. Out of 440 participants, approximately half reported regular exposure to direct-to-consumer ads and deemed them informative. Nonetheless, a substantial number of respondents raised concerns about these advertisements potentially fostering unrealistic expectations regarding the effectiveness of medications. The concern is further amplified by the significant influence of celebrity endorsements, acknowledged by 60.7% of participants as having a major impact on their perceptions. This underscores important ethical considerations regarding the role of celebrities in pharmaceutical marketing and its potential consequences for public health. These findings are in line with existing literature on public attitudes toward DTCA [15,17]. DTCA carries some risks even when it is informative; it can foster unrealistic expectations among consumers and create conflicts of interest for healthcare providers [18,19]. Our study also revealed that consumers demand more detailed information in drug advertisements, including non-medical alternatives, lifestyle changes, and full disclosure of risks and side effects, reflecting a broader public demand for transparency and a holistic approach to medication communication. Several studies highlighted that DTC advertising could misinform patients by overemphasizing benefits, underemphasizing risks, and promoting drugs over healthy lifestyle choices, leading to overutilization and inappropriate prescribing [20,21]. To address social media-based DTCA, regulatory agencies should work on a regulatory framework that focuses on balanced, accurate, and non-misleading advertising communications, requiring manufacturers to disclose benefit and risk information, responding to off-label information requests, and correcting misinformation about products [22]. While over one-third of the participants are aware of the regulations set by the Saudi Food and Drug Authority (SFDA), a significant 82.3% believe it is acceptable to advertise medications within these regulations. This indicates a gap between the awareness of regulations and perceptions of their adequacy, underscoring the need for clear and effective communication about regulatory frameworks [15,22]. 

Customers’ perceptions of pharmaceutical advertisements in Saudi Arabia are also influenced by the quality of the content, the credibility of the source, and the presence of information about adverse effects. These findings point to a public preference for authenticity and factual content in marketing, such as real success stories and experiences. Other studies have shown that consumer-targeted drug advertising can sometimes contain misleading information, contradicting the primary purpose of drug promotion, which is to inform consumers about pharmaceutical products [16,23]. The results of the study also showed that 93.2% of the participants demanded stricter controls on drug advertisements on social media emphasizing the need for enhanced oversight to ensure the accuracy and reliability of information disseminated in these digital platforms. Customers’ demand for strict regulation may be due to social media’s influence on drug marketing and the resulting distrust towards the information presented [22,24]. The SFDA has clear policies in place for penalizing companies or online advertisers that violate advertising regulations, including fines and other punitive measures for non-compliance with their strict guidelines. A potential limitation of our study is the sample’s age range, which may not fully capture the diverse perspectives across all age groups. Additionally, while the convenience sampling method was used, it may not represent the entire population, potentially influencing the study’s generalizability. A large portion of the participants in this study had a bachelor’s degree. This overrepresentation of educated individuals may affect the generalizability of the results, as it might not reflect the views of the wider public. This study sheds light on the intricate nature of pharmaceutical advertising in Saudi Arabia, emphasizing the need for more ethical marketing strategies, ensuring advertising practices align with public expectations and needs while adhering to strict regulatory standards to protect public health and trust. Future research should evaluate the effectiveness of current regulatory frameworks and explore consumer reactions to different types of drug advertising across various media platforms. Investigating methods to enhance public trust and ensure transparency in drug advertisements will be key to developing more ethical advertising practices. Strengthening oversight and improving communication about regulations are essential to aligning advertising practices with public expectations and safeguarding public health.

## 5. Conclusions

Pharmaceutical advertising must strike a balance between clear communication and education. The public expects straightforward, accurate details, especially on social media, where tighter regulations are essential. Collaboration with pharmaceutical companies is vital to ensure ads reflect health guidelines. Key actions include stronger oversight of digital ads and raising public awareness about the limitations and risks of these advertisements.

## Figures and Tables

**Table 1:** Demographic characteristics of studied participants.

Items	Studied Participants. (*n* = 440)	
	No	%
Gender		
Male	215	48.9%
Female	225	51.1%
Age		
Mean ± SD	33.83 ± 12.05	
Range	18–65	
Nationality		
Saudi	425	96.6%
Non-Saudi	15	3.4%
Province		
Riyadh province	23	5.2%
Makkah province	375	85.2%
The Eastern province	12	2.7%
The Southern province	13	3.0%
The Northern province	12	2.7%
Medina Al-Munawwarah province	5	1.2%
Marital status		
Single	205	46.6%
Married	204	46.4%
Divorced	17	3.8%
Widowed	4	0.9%
Prefer not to disclose	10	2.3%
Education level		
Primary school	4	0.9%
Middle school	6	1.4%
Secondary school	83	18.9%
Bachelor’s degree	313	71.1%
Diploma	5	1.1%
Master’s degree	24	5.5%
Doctorate degree	3	0.6%
Others	2	0.5%
Current occupation		
Employed	225	51.1%
Unemployed	53	12.0%
Student	128	29.1%
Retired	22	5.0%
Self-employed	9	2.1%
Others	3	0.7%
Family Income		
Less than 6000 SAR	76	17.3%
Between 6000 to 15,000 SAR	185	42.0%
Between 15,000 to 30,000 SAR	91	20.7%
Between 30,000 to 50,000 SAR	13	3.0%
More than 50,000 SAR	5	1.1%
Prefer not to disclose	70	15.9%

**Table 2:** Awareness and attitudes of participants towards drug advertisements.

	Studied Participants. (*n* = 440)	
	No	%
How often do you notice drug advertisements?		
Very frequently	100	22.7%
Frequently	112	25.5%
Occasionally	155	35.2%
Rarely	67	15.2%
Never	6	1.4%
Do you think drug advertisements are informative?		
Very informative	80	18.2%
Informative	99	22.5%
Neutral	172	39.1%
Not very informative	78	17.7%
Not informative at all	11	2.5%
Have you or someone you know been influenced by a drug advertisement to discuss a specific medication with a healthcare professional or to inquire about a drug?		
Yes	212	48.2%
No	228	51.8%
Do you think you will ever be influenced by a drug advertisement? If so, how do you think it will impact your decision-making?		
Yes, it will strongly influence my decision.	93	21.1%
Yes, it will somewhat influence my decision.	232	52.7%
No, I will not be influenced.	115	26.2%
Do you think drug advertisements will create unrealistic expectations about the effectiveness of certain medications?		
Yes, often.	154	35.0%
Sometimes, it depends.	241	54.8%
No, not really.	45	10.2%
What are your thoughts on the use of celebrity endorsements in drug advertisements? Do you think it will influence people’s perceptions?		
Yes, it will influence people’s perceptions.	267	60.7%
No, it won’t have much influence.	66	15.0%
I’m not sure, it could go either way.	107	24.3%
Do you think drug advertisements should be required to include more information about nonmedical alternatives or lifestyle changes for treating certain conditions?		
Yes, it’s important to provide that information.	377	85.7%
No, it’s not necessary	63	14.3%
Should drug advertisements provide enough information about potential risks and side effects?		
Yes, they should provide more information.	401	91.1%
No, people may avoid using the drug.	39	8.9%

**Table 3:** Participants’ opinions on drug advertisements and the reasons behind those opinions.

	Studied Participants. (*n* = 440)	
	No	%
Please select your level of agreement with the following statement: “I believe that drug advertisements should be allowed in public spaces (e.g., on billboards or at bus stops)”.		
Strongly agree	70	15.9%
Agree	123	28.0%
Neutral	120	27.3%
Disagree	75	17.0%
Strongly disagree	52	11.8%
Please select your level of agreement with the following statement: “I believe that drug advertisements should be allowed and reach to mobile phone or other personal devices (e.g., apps, websites)”.		
Strongly agree	71	16.1%
Agree	120	27.3%
Neutral	102	23.2%
Disagree	86	19.5%
Strongly disagree	61	13.9%
If you agree with drug advertisements in public spaces or on personal devices, please choose one of the reasons why.		
It educates people about available treatments.	218	49.5%
It encourages people to seek help for illnesses they may not have known about.	170	38.6%
Drug advertising is a valuable source of information that raises awareness of medications.	136	30.9%
If you disagree with drug advertisements in public spaces or on personal devices, please explain your reasons briefly.		
It spreads misleading Information	139	31.6%
It pressures healthcare providers to prescribe specific medication that the patient saw in advertisements, or the patient won’t take it.	134	30.5%
It contributes to overmedication by encouraging people to seek medical solutions to minor or normal health problems that could be treated without medication.	242	55.0%
Some medications are not suitable for all patients, so it is difficult to change patients’ minds if they are convinced of this medication	2	0.5%
Abolition of the role of education by physician	1	0.2%

**Table 4:** Participants’ opinions on additional insights regarding preferred advertising platforms, legal aspects, credibility, and experiences with drug advertisements.

	Studied Participants. (*n* = 440)	
	No	%
Which of the following advertising mediums do you prefer for drug advertisements?		
Billboards	165	37.5%
Mobile apps and websites	207	47.0%
Social media	204	46.4%
Television and radio	104	23.6%
Magazines and newspapers	47	10.7%
None of the above	117	26.6%
Are you aware of SFDA regulations regarding drug advertising		
No	292	66.4%
Yes	148	33.6%
Do you think it is permissible to advertise medicines in accordance with the laws and regulations issued by the Food and Drug Authority in the Kingdom of Saudi Arabia?		
Yes, it is permissible according to laws and regulations	362	82.3%
No, this is not permissible according to laws and regulations	78	17.7%
What is the main factor that affects your overall impression of pharmaceutical advertisements?		
Credibility of the source	344	78.2%
Quality of content	191	43.4%
Celebrity influence	65	14.8%
Providing information about side effects and risks	245	55.7%
The design of the ad itself	64	14.5%
Do you prefer to see drug advertisements that show success stories or real-life experiences of people?		
Yes	298	67.7%
No	142	32.3%
How do you think the quality of pharmaceutical advertising can be improved?		
Increased focus on medical and scientific information	300	68.2%
Providing more awareness about the safe use of medications	263	59.8%
Discuss non-pharmacological alternatives to treatment	177	40.2%
Provide real-life examples and success stories	159	36.1%
Increase the transparency of information about risks and side effects	214	48.6%
Do you think there should be stricter rules for pharmaceutical advertising?		
Yes	68	15.5%
No	372	84.5%

## Data Availability

The data presented in this study are available on request from the corresponding author. The data are not publicly available due to privacy considerations and ethical guidelines.
